# Simulating MR imaging for the human embryonic heart

**DOI:** 10.1186/1532-429X-17-S1-P48

**Published:** 2015-02-03

**Authors:** Georgios Kantasis, Christos G Xanthis, Anthony H Aletras

**Affiliations:** 1 Cardiac MR group Lund, Dept. of Clinical Physiology, Lund University, Lund, Sweden; 2 Computer Science and Biomedical Informatics, University of Thessaly, Lamia, Greece

## Background

MRI has evolved to become a safe diagnostic imaging tool for prenatal care. Recent studies have demonstrated its applications in early diagnosis of congenital heart anomalies (Loomba et al. 2011). However, in vivo embryonic MRI presents obstacles associated with small sample size, RF field profile distortion, RF exposure limits, motion etc. Therefore, small structures such as the heart are difficult to image.

In clinical practice during the first trimester, MRI scans are reserved only for severe cases and research protocols are not easily approved. Optimization of pulse sequences is not a trivial issue and exploring the entire parameter space is impractical. For this reason simulations of MRI pulse sequences and imaging protocols can be utilized on embryonic and fetal anatomical models. In this study, a simulation framework is presented that allows MR analysis of the embryonic heart in order to investigate the limitations of image resolution and quality.

## Methods

A detailed anatomical model was developed, based on the Multi-Dimensional Human Embryo dataset (Smith et al. 1999). The dataset consisted of 38 transverse slices with Diffusion, T1 weighted and T2 weighted images, acquired at 9.4T. The dataset consisted of a 23^rd^ Carnegie stage (approximately 56 days) fixed embryo, with a total of 38 slices and an isotropic voxel size of 156.3μm^3^. A semi-automatic segmentation algorithm was applied to obtain the tissue masks and a 3D model of the human embryo was developed.

**Figure 1:**
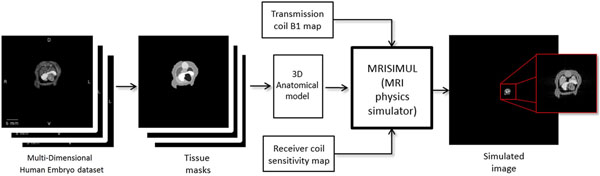
The block diagram of the method used. In the true MR images we can see the heart and surrounding tissues. The next image shows the tissue masks of the recognized structures within the body of the embryo. The image acquired as a result of the simulation can be seen in the right. Notice the low resolution of the region of interest and its small size relative to the FOV.

A recently developed GPU-based (Graphics Processing Units), comprehensive Bloch equation simulator of MR physics was utilized (Xanthis et al. 2014). A Gradient Echo (GE) pulse sequence was applied to the 3-D object with TR=8ms, RF duration=2ms, flip angle=15°, acquisition matrix=400 x 400 and FOV 200mm x 200mm (B_0_=1.5T). The B1 transmission profile and the receiver coil sensitivity map were simulated using an exponential attenuation function as described by (Wen et al. 1997). A block diagram of the method is shown in Figure 1.

## Results

**Figure 2:**
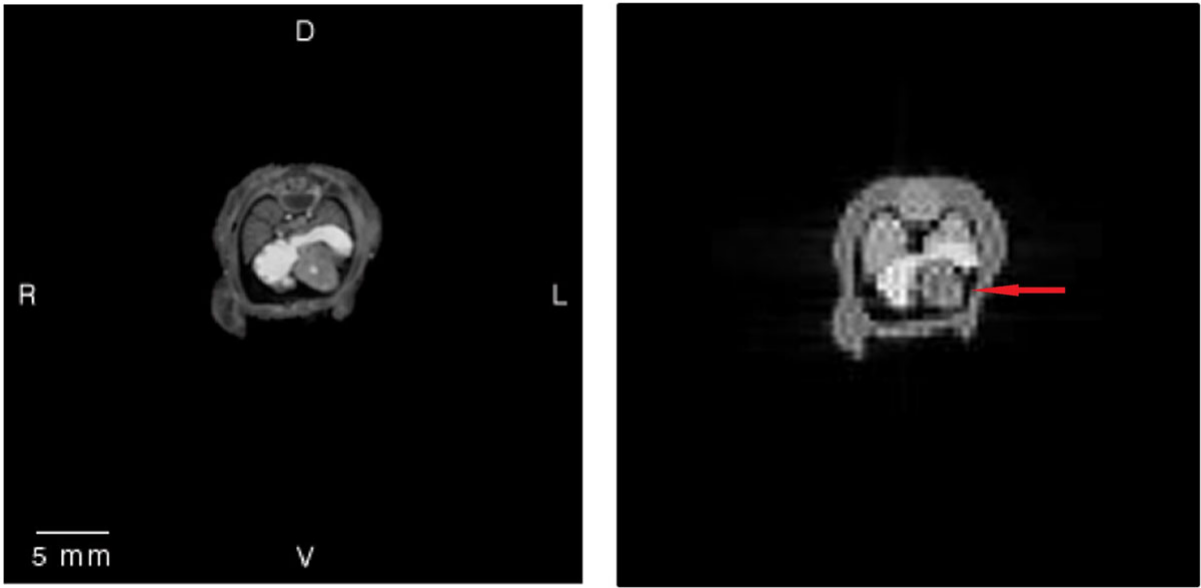
Left, the Multi-Dimensional Human Embryo dataset. Right, the acquired image from the simulator. The loss in resolution is evident and the heart is difficult to delineate (arrow).

A simulated tomographic image of the anatomical model is presented in Figure 2. The large FOV required to image the uterus, the low resolution (pixel size=0.5mm^2^), the B1 profile and the receiver coil sensitivity may limit differentiation of the heart from the surrounding tissues.

## Conclusions

In this study we presented a simulation framework which can allow for fetal and embryonic MRI to overcome its obstacles through the design of appropriate pulse sequences and protocols.

## Funding

Funding provided by the Greek Excellence Program (ARISTEIA I).

